# I’m Not That Person: A Qualitative Study of Moral Injury in Forensic Psychiatric Patients

**DOI:** 10.3390/ijerph22030372

**Published:** 2025-03-03

**Authors:** Sarah K. Atkey, Krystle Martin, Karen D. Fergus, Joel O. Goldberg

**Affiliations:** 1 Department of Psychology, Faculty of Health, York University, Toronto, ON M3J 1P3, Canada; sdewdney@yorku.ca (S.K.A.); dr.krystle.martin@gmail.com (K.M.); kfergus@yorku.ca (K.D.F.); 2 Department of Psychology, Faculty of Social Sciences and Humanities, University of Ontario Institute of Technology, Oshawa, ON L1G 0C5, Canada

**Keywords:** moral injury, forensic psychiatric patients, violence, traumatic stress

## Abstract

Few studies have examined how committing criminal acts of violence impacts the lives of perpetrators who were mentally ill at the time of offence and in which the act itself reflects behaviour that is uncharacteristic of the individual. Theoretical accounts and clinical reports describe a phenomenon termed moral injury, which profiles the deleterious emotional effects that can arise from actions that transgress moral beliefs and expectations. Shame, guilt, spiritual/existential conflict, and loss of trust are considered to be core symptoms of moral injury with growing empirical studies which examine moral injury in military and public safety worker samples. The extent to which these kinds of moral injury phenomena might be evident among mentally ill perpetrators was explored using a qualitative-methods approach in a sample of 19 adult participants hospitalized in a Canadian forensic programme inpatient service. The sample consisted of 13 male and 6 female patients, with a mean age of 36.2 years (*SD* = 10.8), and the majority diagnosed with schizophrenia or schizoaffective disorder. A qualitative interview was conducted where participants were asked to describe feelings about the index offence, the effect it has had on their well-being, and how they have coped with having committed the offence. Using a reflexive thematic analysis process, 5 themes and 23 subthemes were generated that relate to the various resultant impacts. The five themes which emerged were (1) Living with the Emotional Aftermath; (2) Trying to Make Sense and Coming to Terms; (3) My Eyes Have Opened; (4) Facing the Music; and (5) Moving On. The findings are discussed in terms of their implications for understanding forensic inpatients who may be attempting to come to terms with violence they committed while mentally ill and for informing moral injury intervention strategies which might be adapted for forensic mental health services and public health recidivism prevention programmes.

## 1. Introduction

There is a paucity of qualitative research examining the subjective experiences of forensic psychiatric patients, and, in particular, those who committed violent offences [1]. As a group, their voices have often not been heard due to stigmatization [2]. It seems important to gain a deeper understanding of the experiences of hospitalized mentally ill violent offenders given the involuntary nature of much of their treatment and ensuing restrictions on their freedom.

Many of the qualitative studies conducted with forensic psychiatric patients to date have examined their accounts of recovery [3,4,5,6,7,8,9,10]. A meta-synthesis of qualitative recovery research revealed three overarching themes: safety and security as a necessary base for the recovery process, the dynamics of hope and social networks in supporting the recovery process, and work on identity as a changing feature in the recovery process [11]. Recovery in this population is unique as they are not only recovering from severe mental illness, but also from their offences while living under the auspices of the review boards in the forensic psychiatry system.

Closely related to those examining recovery, several studies investigated forensic patients’ experiences of their offences [5,12,13,14,15]. Adshead and colleagues [12] posit that the processes of the patients providing a narrative of their offences and their self-reflection in terms of their identity are important to the understanding of recovery in forensic treatment. A study investigating forensic patients’ experiences of their offences and the meaning they have given them concluded that the narratives fell into one of three groups: criminal stories, victimization stories, and recovery stories [13]. Askola and colleagues [14] concluded that the processing of the offence and the factors leading up to and relating to the offence is an integral part of forensic treatment. Further, a UK study of forensic patients produced themes that emphasize the importance of their offence narratives in their search for meaning [5].

Work by O’Donahoo and Simmonds [16] exploring remorse in forensic patients demonstrated that while the majority of individuals experienced remorse, expressing their remorse was extremely challenging due to painful memories of the offence, its consequences, and its irreversibility. The participants indicated that active symptoms, issues related to insight, and a hesitancy to be unguarded with mental health professionals were identified barriers from communicating their remorse. There is growing body of literature demonstrating findings of post-traumatic stress disorder (PTSD) arising amidst non-legally sanctioned violence and homicide in correctional and forensic populations, with the existing literature suggesting that the prevalence rate of offence-related PTSD in correctional populations is between 15% and 43% [17,18,19,20,21]. Among the public health implications is that PTSD has been associated with future risk of aggressive behaviour and criminal recidivism in correctional populations [22,23].

Moral injury has been described as the long-term deleterious emotional, psychological, behavioural, spiritual, and social effects that perpetrating, failing to prevent, or bearing witness to acts that transgress deeply held moral beliefs and expectations may have on an individual [24,25]. In his syndromal definition of moral injury, Jinkerson [26] describes the core symptoms of shame, guilt, spiritual/existential conflict, and loss of trust, while depression, anxiety, anger, re-experiencing, self-harm, and social problems are considered to be secondary symptoms. Several qualitative studies have examined the experience of trauma caused by committing the offence in forensic patients [27,28,29]. Investigations conducted by Maddocks [27] and by Roth and colleagues [29] discovered themes suggestive of moral injury in forensic patients through methodology that used interviews with both patients and staff. Specifically, Maddocks [27] examined individuals who had received mental health treatment and were convicted of violent offences but the offences were not necessarily committed while the individuals were mentally unwell. The results demonstrated that the participants experienced moral injury as a result of their offending in addition to describing other potentially morally injurious events including adverse childhood experiences and aspects of their treatment in the criminal justice system. A Canadian study [29] interviewed forensic psychiatric patients who were determined to be Not Criminally Responsible on Account of Mental Disorder (NCRMD), as well as staff members on the patients’ hospital units. The results suggested that patients experienced symptoms of moral injury such as guilt, shame, and a loss of trust in their own morality. Finally, an interpretive phenomenological analysis of interviews with two forensic patients with diagnoses of PTSD who had completed treatment for offence-related trauma produced two superordinate themes related to their experiences of trauma [28]. The first theme, journey to forgiveness, described an ongoing journey toward self-forgiveness with obstacles to overcome along the way. The second theme, living with the whole me, depicted the participants’ experience of a “fragmented sense of self” [28] resulting from committing the offence. Participants described the need to accept and reconcile both parts of themselves to “feel whole again” [28].

The studies described above provide insight into the subjective experiences of forensic psychiatric patients in terms of recovery, how they experienced their offences and resultant trauma. A broad examination of the impact of the offence on the ways in which forensic patients think and feel about themselves and the world around them, and the ways in which they cope with these impacts is lacking in the existing literature. Given the lack of power that patients in the forensic system possess, engaging in research to determine their experiences of their offences is an essential step towards providing the best public healthcare and treatment for this vulnerable population. This study proposes to expand our knowledge with novel findings regarding how committing an offence impacts mentally ill forensic patients, exploring their emotional worlds.

In terms of the goals of the current study, we examined individuals who were found NCRMD and committed criminal acts while acutely mentally ill; we consider that for at least some of these individuals there may be ensuing recognition that committing the offence(s) may have transgressed their own deeply held moral beliefs and so may suffer from moral injury. The main objective of this qualitative method study is to explore the potential for the presence of moral injury phenomena among forensic patients by examining their cognitions and affect through their self-reflections regarding their index offence experience. Further, we are interested more broadly in the ways in which the index offence has impacted forensic patients and how they have come to cope with those psychological impacts on their lives to better inform public healthcare and treatment.

## 2. Materials and Methods

### 2.1. Participants

Participants consisted of 19 adult patients receiving inpatient services from the forensic programme at a Canadian public teaching hospital providing assessment and treatment services to individuals living with serious and complex mental illness. Inclusion criteria included having an oral proficiency in the English language and having committed a violent offence with identifiable victims who likely would have experienced physical or psychological harm (murder, attempted murder, manslaughter, assault, etc.) in the past ten years. Only those patients who were deemed to be capable of consenting were included in the study.

### 2.2. Procedure

For recruitment, a combination purposeful sampling strategy was used, comprising snowball and convenience methods [30]. Participants were recruited via advertisements on the inpatient forensic units and by a member of the research team who attended community meetings on the units to describe the study and answer any questions. Patients indicated their interest in the study and were advised that a member of the research team would return to the unit at a later date to facilitate their participation. Patients were also able to contact the researchers directly should they wish to participate. Other recruitment strategies included asking unit staff to suggest patients who may be interested in participating in the study and approaching these patients on the units. Recruitment and meetings with interested individuals took place within a span of two weeks. All individuals who met with the researcher agreed to participate in the study, though as noted below, six individuals were not included in the study sample.

The first author (S.K.A.) met with interested potential participants individually on the units to explain the study in more detail, answer any questions, conduct the informed consent process, and complete the study protocols. All interviews were conducted by the first author (S.K.A.). The interview questions are listed in Appendix A. The first author (S.K.A.) had previous clinical contact with three of the participants (two in a group format, one in individual therapy) several years prior to the data collection during a placement at the site. Participants were informed that the interview would take approximately 60 min and the questionnaires would take approximately 30 min. Participants were compensated CAD 10 for their time. Participants were not pre-screened prior to the interview for ineligibility based on the recency or the level of violence of their offence criteria. All otherwise eligible and interested participants participated in the interview and their eligibility was determined post hoc. Interviews were conducted with 25 participants. Two participants were deemed ineligible based on low levels of physical violence or seriousness of their offences and/or not having an identifiable victim (uttering threats and threaten to burn property; flight from police, theft over CAD 5000, breach probation, and driving while disqualified) and four participants were not included for not answering the interview questions. The data provided by the aforementioned six participants were removed from the dataset.

Interviews were semi-structured and comprised follow-up questions and prompts in addition to the prepared questions in order to obtain detail, depth, and nuance in the answers [31] and any clarification needed. Participants were asked to describe the index offence’s meaning in their lives. Questions probed the participants’ feelings about the index offence, the effect it has had on their well-being, and how they have coped with having committed the offence. Participants were not asked explicitly about moral injury or moral injury symptoms (i.e., shame, guilt, spiritual/existential conflict, and loss of trust) but were instead asked about emotions produced by their offences to ascertain whether moral injury symptoms would arise spontaneously. The interviews lasted between six and 126 min (*M* = 25.4, *SD* = 25.8). The interviews were audiotaped and subsequently transcribed verbatim. After the interviews were completed, the participants were asked to complete the Post Traumatic Stress Disorder Checklist for DSM-5 (PCL-5) [32] among other measures not reported here, as part of a broader project.

### 2.3. Measures

Demographic information was collected with participant permission from the electronic health records including gender, year of birth, mental health diagnoses, index offences, and year of index offences. There were a number of questionnaires administered but for the purpose of this study, only the PTSD measure is reported.

PTSD was measured using the PCL-5 [32]. The PCL-5 is a self-report scale that assesses the presence and severity of PTSD symptoms based on DSM-5 criteria. It is a 20-item measure utilizing a 5-point Likert scale anchored by 0 = not at all and 4 = extremely. Higher scores on the PCL-5 indicate the increased experiencing of PTSD symptomatology, with total scores ranging from zero to 80. Items on the PCL-5 are based on DSM-5 PTSD criteria [33] and enquire about respondents’ experiences of various problems in the past month in response to a “very stressful experience.” According to the developer of the PCL-5, a cutoff score of 33 on the measure is a reasonable indicator of a provisional PTSD diagnosis [32]. For the current study, participants were instructed to consider their index offences to be the “stressful experience”. In the current sample, the internal consistency was excellent, with Cronbach’s α of 0.96.

### 2.4. Qualitative Analysis

The qualitative analysis was conducted utilizing Braun and Clarke’s [34,35] reflexive thematic analysis method, selected due to its flexibility and accessibility [36,37]. Reflexive thematic analysis highlights patterns of meaning across the dataset, enabling the researcher to “see and make sense of collective or shared meanings and experiences” [36]. Researcher subjectivity and reflexivity are crucial to conducting quality reflexive thematic analysis [34,35,37]. Using this framework, the first author (S.K.A.) operated under the assumption that what the participants relayed to her during the interviews reflected their realities, in that their accounts are an accurate representation of their perceptions and experiences. The first author (S.K.A.) also assumed, however, that the participants’ accounts would be affected by the interactions between the participants and the first author (S.K.A.) during the interview. In addition, through the analysis, interpretation of, and empathic engagement with the text, the first author (S.K.A.) acted as a mediator of the meaning in the data, and as a result, the first author (S.K.A.)’s subjectivity is inherently involved in the process. The first author (S.K.A.) entered the interactions with the assumption that some of the participants may have experienced moral injury as a consequence of their offences. The first author (S.K.A.), a cisgender white woman in her mid-40s, recognizes her position as an outsider researcher (not a member of the population studied) [35], having never been involved in the criminal justice system or diagnosed with a major mental illness. The first author (S.K.A.) has experience working clinically with forensic inpatients. A thorough description of the six recursive phases of analysis (as per Braun and Clarke’s approach [35]) which were used can be found in Appendix A. Producing the report, the final phase of the reflexive thematic analysis process, involved creating a “compelling story about [the] data based on [the] analysis” [37] that addressed the research question. The qualitative data were managed using NVivo R1 software and Microsoft Excel and Word.

## 3. Results

### 3.1. Sample Demographic and Clinical Characteristics

The final sample consisted of 19 participants, of which 13 were male (68%) and six were female (32%). The mean age of participants was 36.2 years (*SD* = 10.8, ranging from 22 to 60 years). In terms of diagnoses, 11 participants had schizophrenia as their primary diagnosis, five participants had schizoaffective disorder (four with bipolar subtype), one participant had intellectual developmental disorder, and one participant had unspecified schizophrenia spectrum and other psychotic disorder as a primary diagnosis. There were high rates of comorbidity within the sample, with 17 participants diagnosed with at least one comorbid disorder, 11 participants diagnosed with at least two comorbid disorders, eight participants with at least three comorbid disorders, six participants with at least four comorbid disorders, and one participant with five comorbid disorders. The participants had a mean number of diagnoses of 3.4 (*SD* = 1.5). Substance use disorders were prevalent, with 15 participants diagnosed with at least one substance use disorder (alcohol use disorder, cannabis use disorder, cocaine use disorder, amphetamine use disorder, opioid use disorder, stimulant use disorder, substance disorder not otherwise specified). Seven participants had been diagnosed with a personality disorder (antisocial personality disorder, personality disorder not otherwise specified). Other comorbid disorders included autism spectrum disorder, attention-deficit/hyperactivity disorder, conduct disorder, and intellectual disability. Interestingly, only one participant had a formal diagnosis of PTSD. Violent index offences ranged in severity from threaten death to first degree murder. Participants had committed between one and 10 index offences with a mean number of offences of 4.6 (*SD* = 2.5). Not all index offences were violent, with non-violent offences including breach of recognizance, failure to comply with probation, flight from peace officer, mischief, and possession of a dangerous weapon. Each participant had at least one violent index offence (which was an inclusion criterion). Age and clinical diagnostic, and other demographic data were unavailable for one participant.

In terms of PTSD levels, the PCL-5 findings showed mean scores of 21.2 (*SD* = 20.4) with a range of 1–65. Using a cutoff score of 33, five of the participants met criteria for a provisional PTSD diagnosis. The only participant with an official PTSD diagnosis in their electronic health record had a score of 40 on the PCL-5. Although interpretation of severity levels has not been established for this measure, based on the rating scales, the overall findings suggest at least mild to moderate self-reported traumatic stress in the sample.

### 3.2. Qualitative Findings

The goal of the qualitative analysis was to determine the presence of moral injury in forensic patient accounts by examining their cognitions and affect regarding their index offence, the impact of the offence on their lives, and their coping strategies. Due to the broad interview questions, the data collected were rich with the participants’ heterogeneous experiences of the offences as life-altering events and the writer (S.K.A.) believed she would be remiss to focus solely on the experiences linked to moral injury in the analysis and reporting phases of the study. During the reflexive thematic analysis process, five themes and 23 subthemes were generated from 19 participant interviews. Each theme relates to the various impacts, emotions, and cognitions experienced by the participants as a result of the index offence. For brevity, this paper will only describe the main themes.

The five themes are (1) Living with the Emotional Aftermath; (2) Trying to Make Sense and Coming to Terms; (3) My Eyes Have Opened; (4) Facing the Music; and (5) Moving on, as well as 23 subthemes subsumed as noted in Table 1 below. Following the recommendation of Braun and Clarke [35], numerical frequencies of the themes and subthemes will not be provided. The themes and subthemes are displayed in Table 1.

#### 3.2.1. Living with the Emotional Aftermath

Almost all participants reported that they have felt a plethora of unpleasant emotions that have negatively impacted their lives after their index offences, and that they are now living with the emotional aftermath of their offences. Participants stated that these emotions are challenging to cope with: “It was just like, very difficult for me to deal with all the emotions like, to do with my offence”. This sense of an emotional aftermath stays with the participants long after the offences occur and cause further suffering. One participant stated, “Sometimes I have to relive the assault where the police come to arrest me for the assault causing bodily harm” while another one noted “Like, I never stop thinking about that day, you know?”.

Participants frequently endorsed experiencing shame, embarrassment, guilt, and regret after their offences. Initially shame, guilt, and regret were treated as three separate subthemes; however, it became clear that there was too much overlap between the subthemes for them to be treated separately. One participant stated “I still feel guilty about it… I wish it never happened”. Another described her feelings by saying, “there’s a lot of guilt and a lot of sadness and grief surrounding it. Like I feel terrible about having done that”. When asked about their feelings about their offences, one participant indicated he felt “down” while another stated she felt “bad and sad”. Many participants expressed remorse over the offences, with one stating about his offence “[it’s] something I could, like, I wish I could take back” and another stating “I hate the fact that it happened”. A particularly guarded participant stated “Uh, the best thing I can say it’s an unfortunate event that transpired and um, I feel horrible and wish events did not take place, but they did”.

#### 3.2.2. Trying to Make Sense and Coming to Terms

Many participants described a period after their offence during which they examined the lead-up to their offences in an attempt to discover why they committed their offences. They indicated that they questioned themselves. One participant stated “And I’m like, why would you do that?”. Another described the offence and its ramifications as “a learning experience” wherein he discovered more about his illness: “I’m just, just understanding more about like my mental health…So, maybe it’s like a rough lesson”.

#### 3.2.3. My Eyes Have Opened

Several participants expressed that they experienced an awakening of some sort in the aftermath of their offences, having been exposed to situations not experienced by them previously, such as jail and the court system. Some participants expressed this ‘opening of their eyes’ as a loss of naivety and a subsequently felt need to be more aware and less gullible in order to protect themselves, as one participant states: “I can’t be as careless as I was before, you know, or as naïve, I’ll say naïve. I can’t be as naïve as I was before. I just have to be like, um, I guess more vigilant… You know, not everything is, not everybody is as they appear to be and not everything is as it appears to be”.

#### 3.2.4. Facing the Music

Regardless of whether participants have been able to resolve any unpleasant emotions they have encountered stemming from their index offences such as shame, guilt, and regret, many participants reflected that the offences and the subsequent NCRMD court rulings have an immeasurable impact on their lives. As one participant stated “It just seemed like a small thing at that time, but it’s changed, like, so much of my life”. They are presented with the challenge of facing the music, and now dealing with being under the auspices of the court system.

#### 3.2.5. Moving On

Many participants expressed forward motion in their lives and a desire to move on. For some, this means apologizing and being forgiven; for some it means learning to cope with their mental illness and circumstances; for some it involves using the supports provided; and for some it means growth and recovery. For some participants, forgiveness was an important part of their journey. One participant indicated that in order to deal with his shame and guilt regarding his offence, he prays for God to forgive him. As another participant stated about his mother, who was also his victim, “she forgave me, she still loves me… That’s been like what has kept me, like, sane”.

## 4. Discussion

The themes and subthemes generated provide a rich description of the impact the violent offence had on the participants’ lives, their experiences of moral injury, and how they have coped with the negative impacts of the offence. The reflexive thematic analysis was not intended to solely focus on the potential for moral injury but on the participants’ experiences as a whole and as such many of the themes do not relate to moral injury. Despite this, many of the participants endorsed they had experienced many of the symptoms in Jinkerson’s [26] syndromal definition of moral injury.

The core symptoms of shame, guilt, and the secondary symptoms of anger and anxiety resulting from the offences were endorsed by many of the participants and contained in the theme Living with the Emotional Aftermath. Shame and guilt over the offences were frequently endorsed together and are encapsulated under the subtheme shame, guilt, and regret. It is possible that participants used these terms interchangeably as is a common tendency in both casual and academic discourse. Some participants indicated they also feel shame regarding their illness and hospitalization in addition to or instead of their offences. Shame, guilt, and regret have been found to be common reactions of forensic patients to their offences [8,13,14,16,27,28,29,38,39].

Participants’ expressions of anxiety were included in the shattered and shaken subtheme and included fear about becoming symptomatic again and reoffending in addition to anxiety about the index offence, consistent with previous work [29]. Roth and colleagues [29] reported that their participants also expressed fears of victim retaliation. Anxiety was not part of the themes generated in Maddocks’ [27] study of forensic patients, but the Maddocks reported [27] that forensic patients experience anxiety around developing close relationships, according to staff member accounts. Without using the term moral injury, Adshead and colleagues [12] discuss their participants’ fear and anxiety around the formation of their new identities after their offences, or the process of reconciling who they are with their actions. Askola and colleagues [14] found that forensic patients experienced anxiety while processing their offences, while another study found forensic patients experienced anxiety after their offences about having an uncertain future [9]. Finally, similar to results of the current study, Laithwaite and Gumley [6] reported that participants felt anxious about their ability to cope while living in the community. The data under the subtheme Anger and Frustration depicts participants’ anger directed at themselves, others, and the forensic and criminal justice systems. One participant’s expression of his anger included angry outbursts, throwing objects, and physically assaulting others. Again, these findings are in line with other research examining the impacts of the offences on forensic patients, with anger frequently cited as an effect [5,12,16,27,28,29,40,41].

Just one participant endorsed experiencing the core symptom of spiritual/existential conflict which is subsumed under the theme Trying to Make Sense and Coming to Terms. This participant described a period of spiritual conflict during which he questioned his relationship with God during the period immediately after his offence. It is noted that this participant believed that God instructed him to kill his mother, and the resulting questioning was more about God’s reasons for His instructions than whether the participant still believed in Him, or related to the participant’s morality. Further, while most participants experienced some aspects of moral injury as noted above, the experience of symptoms was not uniform across participants, with some participants strongly endorsing many symptoms of moral injury and others discussing fewer symptoms. Notably, one participant indicated that he experiences no guilt, shame, or regret regarding his offence and does not “give a shit” that the offence occurred.

Beyond exploring moral injury phenomena, another goal of this study was to examine lived experience and ways of coping. A common theme across the participants is that they are Trying to Make Sense and Come to Terms with their offences. Indeed, other studies demonstrate that coming to terms or making sense of their offences is an important part of recovery for forensic patients [3,5,6,8,11,13,14,27,38,42]. It is noted that often the individuals must come to terms not only with their offences, but also with their diagnosis of mental illness [8]. Many participants chose to view their offence as rough lessons whereby they are planning to learn from their experiences so as not to repeat them. Rough lessons, or considering the offence as a learning opportunity, does not appear to be a theme generated in other studies. Many participants in the current study demonstrated that they were finding the positive angle in their offences either by noting the offences could have been worse, or by stating they are now receiving needed treatment for their mental illnesses; these participants tended to endorse fewer moral injury symptoms. Searching for the positive is possibly a coping mechanism for some to aid in dealing with their current circumstances. This desire to find some positive in a generally negative situation has been found in other qualitative research with forensic patients [5,6,7,38] and is a common attribute among individuals who are highly generative [43]. Under the restorative justice subtheme, participants discussed either their desire to apologize or that they were actually able to apologize to their victims; receiving forgiveness has been an important part of their coping and may be important for informing and integrating with public health recidivism prevention programmes. Other research teams have found similar sentiments among forensic patients in terms of the desire to apologize and receive forgiveness [5,7,8,13,15,16,27,29]. Participants frequently discussed the importance of receiving support from various sources, including family and hospital staff, subsequent to their offences. They relied on this social support as an important aspect of their coping. These findings are similar to results of other qualitative studies conducted with forensic patients [4,5,6,7,8,9,11,13,14,16,38,39,40,42,44,45], highlighting the importance of familial and therapeutic support for this population during recovery.

Several limitations are noted in the current study. First, purposeful sampling was used which comprised snowball and convenience methods. It is unknown whether the patients who did not choose to participate in the study are substantially different in terms of experiences of moral injury than those who volunteered. For example, the individuals who volunteered to participate in the study may be those who have experienced lower levels of unpleasant emotions regarding their offences and thus feel more comfortable discussing its emotional ramifications. Cultural data were not available, which should be considered for future studies. However, the sample was heterogeneous in many ways, including levels of insight, severity of offence, and ability or willingness to articulate emotional states. That stated, the population from which the sample was drawn is also likely heterogeneous in similar ways. It is possible and perhaps even likely that the varying levels of insight among the participants and varied offence severity (only one participant had murder as the index offence) served to under-estimate moral injury findings.

## 5. Conclusions

Despite the serious public health risks posed by mentally ill offenders, few studies have examined their lived experience to attain better understandings. This study confirmed the presence of moral injury in forensic psychiatric patients and through their narratives highlighted the damaging impact the offence has had on their lives. It also demonstrated the incredible strength and resilience of this stigmatized population through illuminating their stories which to date have been rarely listened to and which help to truly see the ‘people behind the label’ in forensic psychiatric hospitals. No theme was present across the entire sample, highlighting that among forensic patients there is no standard way to feel, think, or act, and that each individual’s recovery journey is unique. It is notable that there are varying degrees of insight within the forensic patient population; a possibility for future research examining offence-based moral injury may be to conduct the study with participants who have developed significant insight into their mental illness. Compared to male forensic psychiatric patients, female patients are more likely to have charges of murder or attempted murder [46] and may be more susceptible to moral injury as a result. While no gender differences were found in mental health symptoms and substance use in veterans experiencing moral injury [47], male healthcare workers experiencing moral injury were more likely to abuse prescription drugs and alcohol than their female counterparts [48]. It is possible that moral injury presents differently in male and female patients in the forensic psychiatric population, and it may be prudent to examine each gender’s experiences separately. Given that PTSD has been associated with future risk of aggressive behaviour and criminal recidivism in correctional populations [23,24], moral injury may also be associated with recidivism within forensic populations. Research examining the clinical implications of moral injury in terms of recidivism risk in both correctional and forensic populations may be a helpful addition to the area of forensic risk assessment. Effective treatment currently exists for moral injury in the veteran population; consideration should be given for elements of these treatments to be combined with existing (largely narrative-based) treatments for offence-related concerns to directly alleviate harmful moral emotions and moral injury psychological sequelae.

## Figures and Tables

**Table 1:** Themes and Sub-themes.

Theme	Subtheme
Living With the Emotional Aftermath	Shame, Guilt, and Regret
	Anger and Frustration
	Shattered and Shaken
Trying to Make Sense and Coming to Terms	Rough Lessons
	Could Have Been Prevented
	Finding the Positive Angle
	Symptoms are to Blame
	Dissonance
My Eyes Have Opened	
Facing the Music	Injustice
	Loss of Trust
	Loss of Connection
	Diminished Self-Concept
	Stigma and Judgement
	Loss of Freedom
	Sense of Loss
	Life on Hold
Moving On	Restorative Justice
	Receiving Support
	Unaffected
	Learning to Cope
	Resilience
	Focusing on the Future
	Better Times Now

## Data Availability

The datasets presented in this article are not readily available because the requesting source must be affiliated with an academic institution and due to privacy concerns with the qualitative data. Requests to access the datasets should be directed to the corresponding co-author: jgoldber@yorku.ca.

## Appendix A

### Appendix A.1. Interview Questions

Will you please briefly explain what was happening for you during your index offence? For example, how were you feeling? What were you thinking?
How long ago was your offence?
What has and has not changed since your offence?
How have you changed or stayed the same since your offence?
What are your feelings about your offence? What feelings has your offence brought up for you?
Do you think about yourself differently since your offence?
Have your beliefs about the world changed since your offence?
How have you coped with or handled the thoughts and feelings after the offence?
What has your experience of this interview been?

### Appendix A.2. Qualitative Analysis Procedure

Braun and Clarke’s method [35] involves six recursive phases of analysis: (1) familiarizing yourself with your data, (2) coding, (3) generating initial themes, (4) reviewing and developing themes, (5) refining, defining, and naming themes, and (6) producing the report [34,35]. As per Braun and Clarke [35], the first author (S.K.A.) maintained a reflexive journal throughout the data analysis process. The interview recordings were initially imprecisely transcribed by a research assistant. The first author (S.K.A.) then corrected the original transcriptions to produce orthographic transcripts and to refamiliarize herself with the interviews. The first author (S.K.A.) read through each transcript prior to generating any initial codes.

According to Braun and Clarke [35], codes are the “building blocks of analysis” that “capture specific and particular meanings within the dataset” relevant to the research question. Initial codes were generated from the complete dataset by the first author (S.K.A.), whereby a code was applied to all excerpts of the dataset potentially relevant to the research questions using NVivo R1 software to assist with the collating process. It is considered to be good practice in reflexive thematic analysis to have a single individual generating codes [35,37]. The first author (S.K.A.) frequently debriefed the research team throughout the code generation process. Codes evolved throughout the coding process, either expanding to include additional segments of data, becoming more defined, or separating into multiple codes to better differentiate various meanings in the data. A primarily inductive, or bottom-up, approach to data coding was utilized, meaning that the coding was driven by the content of the data rather than existing theoretical constructs [34,35]. Both semantic and latent coding were conducted, depending on the first author (S.K.A.)’s judgement of the most suitable coding method to capture meaning in each particular data segment. As per Braun and Clarke [35,37], semantic and latent codes are not a dichotomy and it is typical for both semantic and latent coding to be used in reflexive thematic analysis. Semantic or descriptive codes normally “stay close to content of the data and to the participants’ meanings and use of language” while latent or conceptual codes “develop meanings that lie beneath the semantic surface of the data” [37].

During the third phase of data analysis, generating initial themes, codes were grouped together by meaning and candidate themes were formed and named. During this phase, some codes were discarded, others were placed into a ‘miscellaneous’ theme to be revisited at a later stage of analysis, and some codes formed candidate themes. Candidate subthemes were created to delineate salient elements of the candidate themes. The coded data extracts were collated within the identified candidate themes and subthemes. A preliminary thematic map was created to assist with visualization. The theme development phase was iterative, with themes and subthemes consistently being refined and renamed. The fourth phase of data analysis, reviewing and developing themes, involved the refinement of the candidate themes and subthemes. During the first portion of this phase, the candidate themes and subthemes were evaluated in relation to the coded data, with some codes being discarded and others being placed under different themes. Candidate themes were also altered during this review in relation to the coded data. The second portion of this phase involved evaluating the candidate themes and subthemes in relation to the dataset as a whole. Significant redevelopment of the themes and subthemes occurred during this stage, with some themes discarded, others amalgamated, and new ones created to ensure that the candidate themes effectively captured the meaning present in the dataset. The thematic map was modified to represent the revised themes. During the refining, defining, and naming themes phase of data analysis, the collated data extracts for each theme and subtheme were revisited and arranged in a way in which the essence of the theme or subtheme was identified. A definition for each theme and subtheme was developed by selecting extracts from the data that illustrated the researchers’ interpretation of the data. An analytic narrative was written for each theme and subtheme explaining the meaning of the data extracts, how they related to the experience of moral injury in the forensic population, and their relation to the other themes. Each theme and subtheme were given an informative and pithy title. Producing the report, the final phase of the reflexive thematic analysis process, involved creating a “compelling story about [the] data based on [the] analysis” [37] that addressed the research question. The qualitative data were managed using NVivo R1 software and Microsoft Excel and Word.
